# Potential role of jugular vein echographic assessment for central venous pressure estimation

**DOI:** 10.1186/cc14251

**Published:** 2015-03-16

**Authors:** P Balsorano, S Romagnoli, A De Gaudio

**Affiliations:** 1 AOUC Careggi, Florence, Italy

## Introduction

Although recognized as a questionable indicator of the intravascular volume, central venous pressure (CVP) is integrated in many therapeutic algorithms for hemodynamic resuscitation of critically ill patients [1]. In an attempt to simplify CVP estimation, several clinical and ultrasonographic approaches have been suggested [2-5]. Nonetheless, the external jugular vein (EJV) circumference and area have not been evaluated. Considering the role of EJV visual assessment in the clinical estimation of CVP, we hypothesized that EJV ultrasound evaluation could be used to reliably estimate CVP.

## Methods

Patients with a CVC placed as part of clinical management were evaluated. EJV and internal jugular vein (IJV) measurements were performed at the left cricoid level. IJV and EJV were visualized in short axis view; diameters, circumferences and areas were obtained at end expiration with simultaneous CVP measurement. Measures were performed by a single trained operator, who was blind to CVP values.

## Results

**Figure 1:**
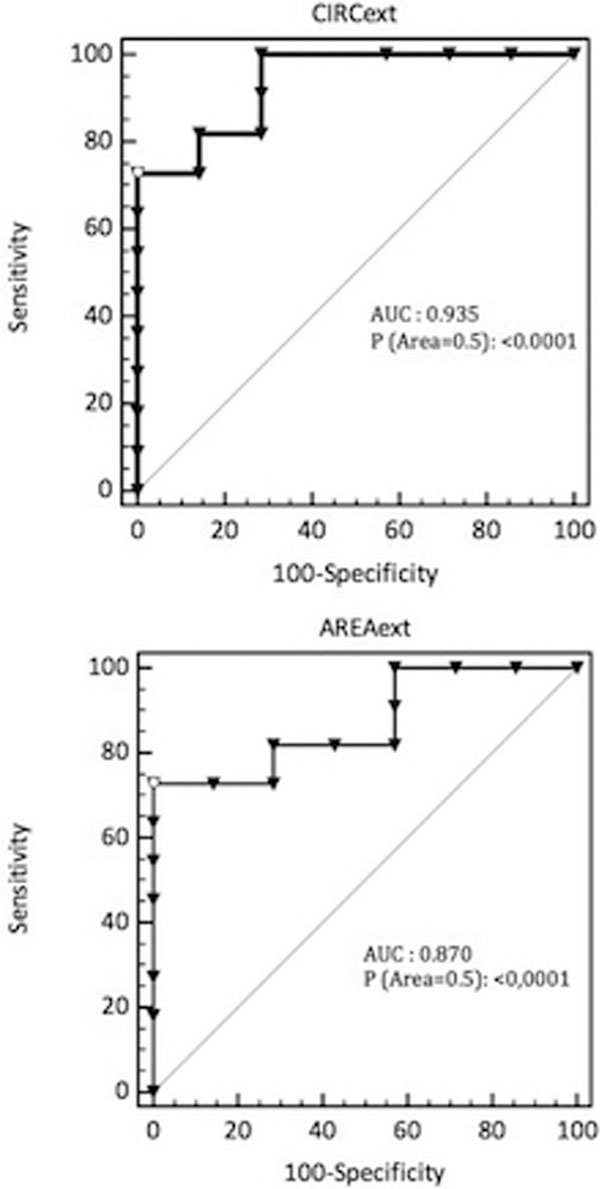


Forty-eight patients were included. A poor correlation was found between CVP and IJV and EJV circumference and area in mechanically ventilated patients. A strong correlation was found between CVP and EJV circumference (*r*: 0.74; *P *= 0.0004; 95% CI: 0.421 to 0.897) and area (*r*: 0.702; *P *= 0.0012; 95% CI: 0.35 to 0.88) in spontaneously breathing patients. Conventional receiver-operating characteristic curves were generated to assess the utility of EJV circumference and area to predict low (≤8 mmHg) versus high (>8 mmHg) CVP values. AUC for EJV circumference and area was 0.935 (*P *< 0.0001; 95% CI: 0.714 to 0.997) and 0.87 (*P *< 0.0001; 95% CI: 0.63 to 0.98) respectively (Figure 1).

## Conclusion

These results highlight a potentially evolving role of EJV circumference and area in the hemodynamic management of spontaneously breathing patients. An important aspect of the suggested approach is its simplicity, requiring basic technical skills and making it suitable in any scenario where an ultrasound machine is available.
